# Variability in Physicochemical Parameters and Its Impact on Microbiological Quality and Occurrence of Foodborne Pathogens in Artisanal Italian Organic Salami

**DOI:** 10.3390/foods12224086

**Published:** 2023-11-10

**Authors:** Frédérique Pasquali, Antonio Valero, Arícia Possas, Alex Lucchi, Cecilia Crippa, Lucia Gambi, Gerardo Manfreda, Alessandra De Cesare

**Affiliations:** 1 Department of Agricultural and Food Sciences, Alma Mater Studiorum—University of Bologna, 40126 Bologna, Italy; frederique.pasquali@unibo.it (F.P.); alex.lucchi3@unibo.it (A.L.); cecilia.crippa2@unibo.it (C.C.); lucia.gambi@gmail.com (L.G.); gerardo.manfreda@unibo.it (G.M.); 2 Department of Food Science and Technology, University of Cordoba, Agrifood Campus of International Excellence ceiA3, Campus Rabanales, 14014 Córdoba, Spain; bt2vadia@uco.es; 3 Department of Veterinary Medical Sciences, Alma Mater Studiorum—University of Bologna, 40126 Bologna, Italy; alessandra.decesare@unibo.it

**Keywords:** pH, water activity, microbial quality, food safety, *Staphylococcus aureus*, *Listeria monocytogenes*, *Klebsiella pneumoniae*

## Abstract

Artisanal salami is produced in small-scale production plants, where the lack of full automation might result in higher variability in food intrinsic properties. The aim of the present study was to evaluate the inter- and intra-batch variability in physicochemical parameters and its impact on microbial quality and occurrence of foodborne pathogens on 480 samples collected from six batches of an artisanal Italian production of organic salami. Relatively high total bacterial counts (TBC) were found on the surface of the table in the stuffing room (4.29 ± 0.40 log cfu/cm^2^). High loads of *Enterobacteriaceae* in the meat mixture of batch 2 and TBC in batch 5 were associated with a higher occurrence of bacterial pathogens. During ripening, water activity (a_w_) and pH failed to reach values lower than 0.86 and 5.3, respectively. Six *Staphylococcus aureus* and four *Listeria monocytogenes* isolates were collected from the salami meat mixture during ripening and the processing environment. A total of 126 isolates of *Enterobacteriaceae* were characterized at a species level, with *Escherichia coli*, *Klebsiella pneumoniae*, *Enterobacter cloacae*, and *Citrobacter freundii* isolated from the final products. Results suggest the relevance of first steps of production in terms of the hygiene of raw materials and handling during stuffing procedures, especially when the physicochemical parameters of the final products do not reach values that represent hurdles for foodborne pathogens.

## 1. Introduction

An increased demand for artisanal foods has been observed in the last decades. These products are generally elaborated locally in small-scale, family-based companies perceived as producing healthier and more ethical food [1,2]. Due to the large variety of local, small-scale productions, microbial data on each specific product are scarce. Moreover, in small-scale food productions, the reduced automation results in variability in physicochemical and microbiological parameters of the final product [3,4].

Italian salami falls within the category of dry fermented sausages with a ripening period longer than 4 weeks and a water activity lower than 0.90 [5]. In Italy, a wide variety of artisanal salami recipes exist with ripening times from 3 to 6 months [6,7]. In traditional salami, the addition of salt and nitrates inhibits Gram-negative spoilage bacteria and enhances coagulase-negative staphylococci (CNS) during fermentation and ripening [7]. CNS use oxygen contributing to a reduction in the redox potential, which in turn inhibits aerobic bacteria in favor of lactic acid bacteria (LAB). Acidification due to LAB growth and the decrease in water activity during natural ripening represent the hurdles reducing the risk of bacterial pathogen growth in fermented sausages [8]. However, the size of these technological hurdles might vary in different productions. In particular, in organic productions, the absence of starter cultures and of nitrates might potentially impact the effective acidification of the food matrix and reduction in Gram-negative and Gram-positive bacterial pathogens. 

Shiga toxin-producing *Escherichia coli* (STEC) as well as *Listeria monocytogenes*, *Salmonella* spp., and *Staphylococcus aureus* were isolated from fermented productions [8,9,10]. *L. monocytogenes* was detected in a French plant producing dry sausages, its equipment, and in 10% of the final products [11]. Higher occurrences of 13–15%, 16%, 42%, and 60% were registered in Italy, Spain, Greece, and Portugal, respectively [2,12,13,14,15]. *Salmonella* was detected in Italy in three dry fermenting processing plants with a prevalence of 16.7% in food and 5.8% in the environment [10]. Additionally, *S. aureus* was detected in raw meat, casing, semifinished, and finished fermented sausages produced in both a nitrite and a nitrite-free production in Spain [9]. Although unusual, outbreaks associated with the consumption of contaminated fermented sausages have been described. *E. coli* O157:H7, O103:H25, and O26:H11 have been identified as etiologic agents in outbreaks involving fermented pork and beef meat salami as vehicles in United States in 1994, in Sweden in 2002, in Italy in 2004, in Norway in 2008, and in Denmark in 2018 [16,17,18,19,20]. Similarly, fermented sausages contaminated by *Salmonella* spp. were associated with outbreaks in Germany in 2001, Spain in 2011, and more recently in the United States in 2022, linked to salami sticks [21,22,23]. 

The aim of the present study was to evaluate the variability in physicochemical parameters and its impact on the microbiological quality and occurrence of foodborne pathogens in an Italian artisanal factory producing an organic pig-meat salami over a year of sampling. Based on results, potential routes of contamination are suggested. 

## 2. Materials and Methods

### 2.1. Experimental Design

**Figure 1:**
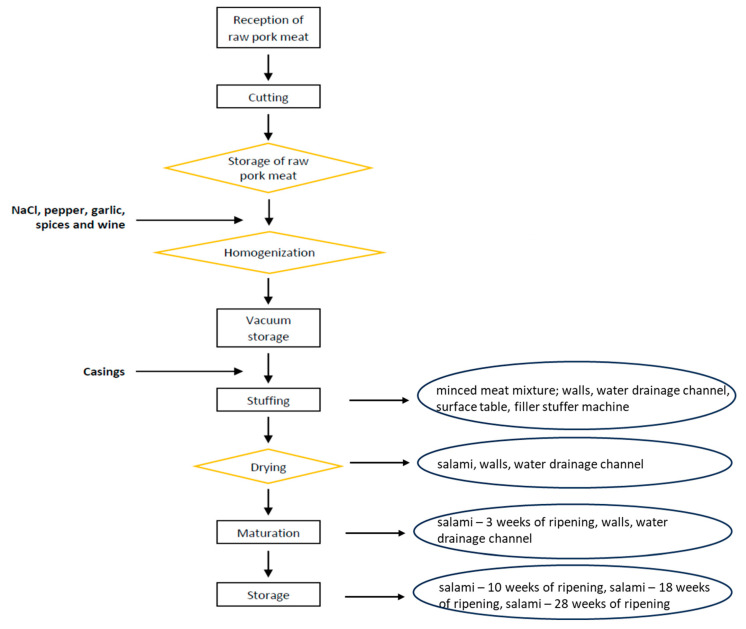
Production flowchart of the Italian organic salami. Sampling spots in the processing environment are indicated in round circles.

One artisanal Italian factory was sampled over 10 months (July 2020–May 2021). The factory produces an organic salami made of pig meat with no addition of nitrites/nitrates or starter cultures and with a ripening period of 6 months. Regarding processing, after the stuffing of a mixture of meat, spices, and salt into natural casing, salami were dried at 10 °C and RH 55–60% for 7–9 days and then stored in controlled environment for 5 weeks at 10 °C and RH 70% for maturation. Afterwards, salami were stored in a cellar up to 6 months (Figure 1). Raw materials were sampled, along with intermediate and final products, as well as the processing environment. A total of 420 samples were analyzed, namely, meat mixture (*n* = 30), salami from the drying room (*n* = 30), salami in the maturation room (*n* = 30), and salami after 10 (*n* = 30), 18 (*n* = 30), and 28 (*n* = 30) weeks of ripening in the storage room. Swabs from the surface of a table in the stuffing room (*n* = 30) as well as the walls (*n* = 90) and drains (*n* = 90) were also collected from the stuffing, drying, and maturation rooms. In addition, swabs from the stuffing machine (*n* = 30) were gathered. For environmental samples, sterile cotton swabs (Copan Italia, Brescia, Italy) were moistened in 10 mL of saline solution (0.9% NaCl) and then used to swab a 100 cm^2^ area. These samples were collected before disinfection and cleaning. Five sample units per matrix (food and environment) per batch were tested. Overall, six batches were analyzed: batch 1 (stuffing on the 1 July 2020), batch 2 (23 September 2020), batch 3 (7 October 2020), batch 4 (21 October 2020), batch 5 (4 November 2020), and batch 6 (18 November 2020).

### 2.2. Microbiological and Physicochemical Analyses

Total bacterial count (TBC) (ISO 4833-2), water activity (ISO 21807), pH (ISO 2917), and the occurrence of *L. monocytogenes* (ISO 11290-1), coagulase positive Staphylococci (ISO 6888-1), verotoxigenic *E. coli* (VTEC) (ISO 16649), and *Salmonella* (ISO 6579) were investigated in all 480 samples [24,25,26,27,28,29,30]. In particular for VTEC, after the isolation of *E. coli* on Tryptone Bile X-GLUC Agar (TBX, Thermo Scientific, Milan, Italy), a PCR for the identification of Shiga toxin-encoding genes was applied as previously described [31]. Additionally, lactic acid bacteria (LAB) (ISO 15214) and *Enterobacteriaceae* (ISO 21528-2) were enumerated in raw materials and semifinished and finished products [32,33]. Moreover, to characterize *Enterobacteriaceae* at a species level, 25 g of food sample was diluted in 225 mL of Buffer Peptone Water (BPW, Thermo Scientific, Milan, Italy) and incubated for 24 h at 37 °C. BPW pre-enriched cultures were then streaked on MacConkey agar (Thermo Scientific) and incubated for 24 h at 37 °C. Five colonies per plate (both lactose fermenting and non-lactose fermenting) were submitted to biochemical test (RapID ONE System and RapID STAPH PLUS System, Thermo Scientific) and PCR for confirmation [31,34,35,36]. One confirmed isolate per species per sample was retained. 

### 2.3. Data Analysis and Modelling

Data analysis and modeling were carried out using R Studio v4.2.2. Statistical comparisons were made between microbial counts from various food batches and environmental samples through ANOVA (with a significance level of *p* ≤ 0.05). To identify homogeneous groups, we applied Tukey’s HSD test. Additionally, we created boxplots to visually represent the variation in microbial counts within and between batches of salami samples stored at different temperatures throughout their shelf life.

Longitudinal data sets, encompassing TBC and *Enterobacteriaceae* counts, collected from various sources such as environmental surfaces, food contact surfaces, meat mixture, and the finished salami within the drying room were analyzed using generalized linear mixed models (GLMMs). These models were adjusted using the R packages *lme4* [37] and *nlme* to account for inter-batch variability, treating it as a random effect. Main effects considered the influence of the variables “Stage” (mixing, stuffing, drying, and ripening) according to the form:

(1) $$Y_{is\left(b\right)}=\left(\beta_{0}+u_{b}\right)+Stage_{s}+\varepsilon_{is\left(b\right)},$$

where $Y_{is\left(b\right)}$ represents the count (in log cfu/g) of a specific microbial group (TBC and *Enterobacteriaceae*), determined within the processing stage denoted as ***s***, belonging to the batch labeled as ***b***; ***β*_0_** is the model intercept which can undergo random shifts denoted as ***u_b_***, associated with the specific batch ***b***; $Stage_{s}$ represents a particular processing stage ***s***; and $\varepsilon_{is\left(b\right)}$ represents the error associated with the microbial count ***i*** determined within the processing stage ***s***, belonging to batch ***b***. Samples, including environmental and drain swabs, meat mixture, and finished salami collected from each processing stage, were integrated into another main effects model as follows:

(2) $$Y_{is\left(b\right)}=\left(\beta_{0}+u_{b}\right)+Stage_{s}\left(Environment_{i}\right)+\varepsilon_{is\left(b\right)},$$

where $Stage_{s}\left(Environment_{i}\right)$ represents a sample denoted as ***i***, which was obtained from the processing stage ***s*** within a specific batch ***b***. The variability between batches within a factory was quantified by assessing the squared standard deviation of the random effects, while it was assumed that errors also conformed to a normal distribution.

## 3. Results

### 3.1. Enumeration of Total Bacterial Count, Lactic Acid Bacteria, and Enterobacteriaceae

**Figure 2:**
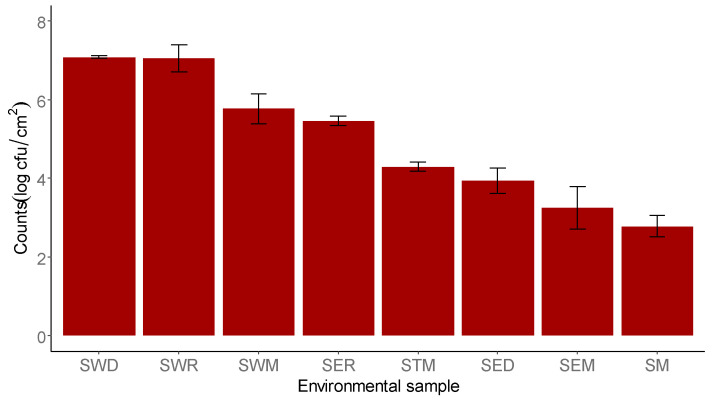
Total bacterial count (TBC) (log cfu/cm^2^) on environmental samples collected at the processing plant. SWD: manhole swab—drying room; SWR: manhole swab—ripening room; SWM: manhole swab—stuffing room; SER: wall swab—ripening room; STM: surface swab—stuffing room; SED: wall swab—drying room; SEM: wall swab—stuffing room; SM: minced-meat machine swab—stuffing room (mean ± standard deviation of 6 batches).

Regarding environmental swabs collected at the processing plant, manhole samples presented the highest loads of TBC compared to the other tested environmental sites (Figure 2). More specifically, manhole samples in the drying and ripening rooms (SWD and SWR) showed the highest TBC, with a mean value of 7.08 ± 0.29 log cfu/cm^2^ and 7.05 ± 0.61 log cfu/cm^2^, respectively. No statistically significant differences (*p* > 0.05) were observed for TBC between batches, also supported by the high intra-batch variability. Interestingly, the surface swab of the table in the stuffing room (STM) showed TBC 4.29 ± 0.40 log cfu/cm^2^ higher than those found in the other swabs of the same room, notably the stuffing machine swab (SM, 2.77 ± 0.99 log cfu/cm^2^) and the wall swab (SEM, 3.24 ± 1.32 log cfu/cm^2^) (Figure 2).

**Figure 3:**
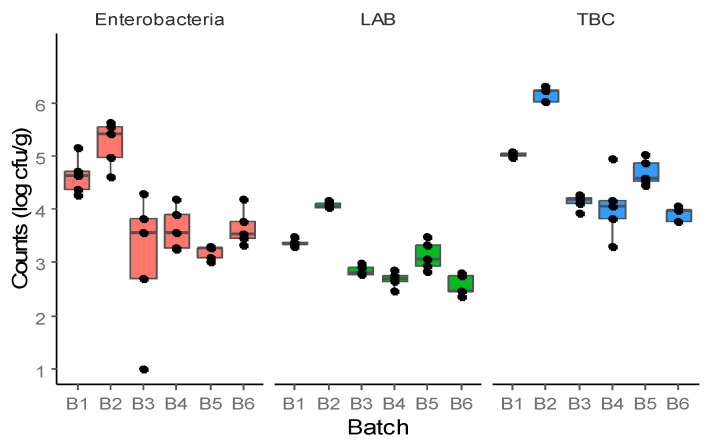
*Enterobacteriaceae*, lactic acid bacteria (LAB), and total bacteria counts (TBC) of meat mixture samples (*n* = 5) belonging to six different batches.

Regarding the meat mixture used to produce salami, high intra- and inter-batch variability for the different groups evaluated was observed, especially for *Enterobacteriaceae* (Figure 3). Higher *Enterobacteriaceae* counts were detected in meat mixture samples of batch 2 compared to the other batches, except for batch 1 (*p* ≤ 0.05). In addition, significantly higher LAB counts were registered in samples of batch 2. Higher TBC counts in the meat mixture samples of batch 5 were observed compared to the other batches, except for batch 2 (*p* ≤ 0.05). 

**Figure 4:**
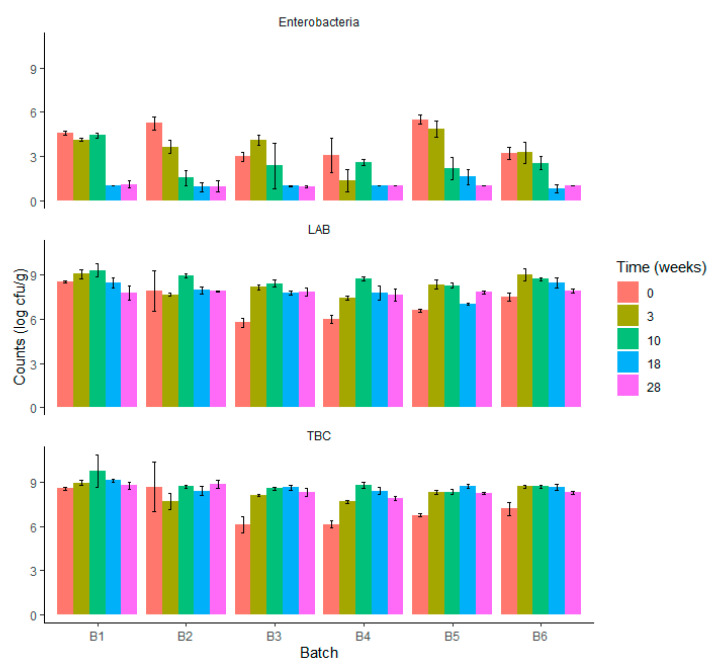
*Enterobacteriaceae*, lactic acid bacteria (LAB), and total bacterial counts (TBC) of salami samples analyzed over the production process: drying room (time = 0) and ripening (3 to 28 weeks) for the six batches evaluated (*n* = 5 for each batch).

Regarding salami samples, slight increases in TBC and LAB counts were associated with a decrease in *Enterobacteriaceae* load in all batches (Figure 4). Interestingly, the increase in TBC and LAB reached maximum levels in salami at 3 or 10 weeks of ripening (depending on the batch), after which a decrease was observed up to 28 weeks of ripening. The increase in TBC in batch 1, for example, started at 8.58 log cfu/g in minced meat mixture and reached 9.75 log cfu/g in salami after 10 weeks of ripening, and slightly decreased to 8.77 log cfu/g in the final product (28 weeks of ripening). Similarly, in batch 1, LAB loads increased from 8.53 to 9.34 log cfu/g (10 weeks of ripening) and reached 8.10 log cfu/g in the final product. Regarding *Enterobacteriaceae*, loads showed a decreasing trend all across the ripening process, starting from 4.56 and reaching 1.09 log cfu/g in the final product in batch 1 (Figure 4). No statistically significant differences were observed between batches in TBC and LAB loads in salami after 28 weeks of ripening (*p* > 0.05). In the final product of all batches, the load of *Enterobacteriaceae* was lower or close to the detection limit of 1 log cfu/g, suggesting that the ripening process was effective in reducing the risk for human health related to the potential occurrence of foodborne pathogens included in this bacterial family. 

### 3.2. Physicochemical Parameters (pH and a_w_)

**Figure 5:**
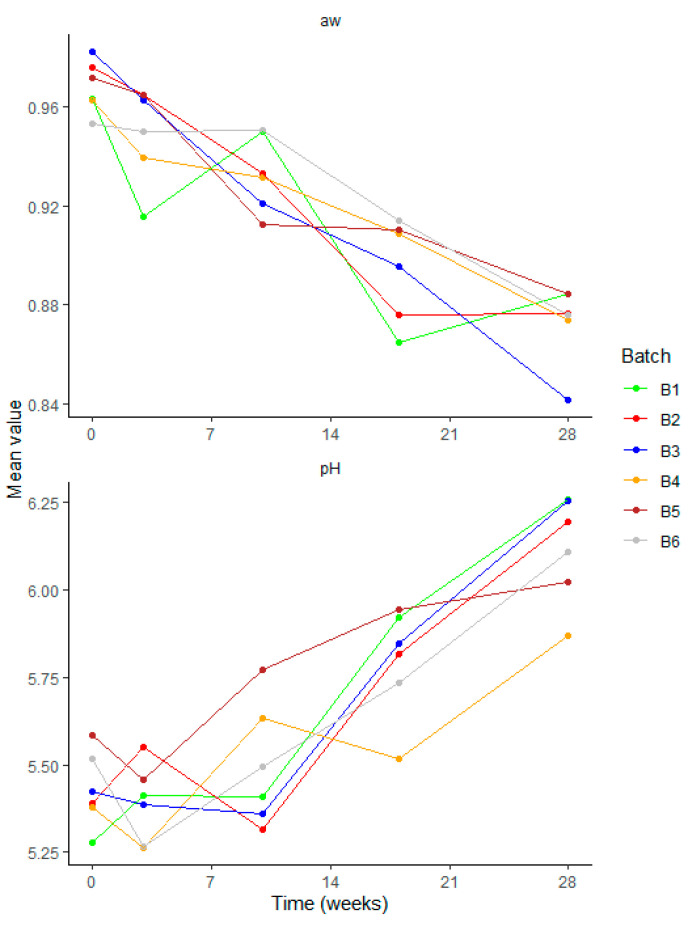
Water activity (a_w_) and pH of salami samples analyzed over the production process: drying room (time = 0) and ripening (3 to 28 weeks) for the six batches evaluated (*n* = 5 for each batch).

As expected, the pH decreased along with the increase in LAB up to 3–10 weeks of ripening, after which it increased along with a reduction in LAB (Figure 4 and Figure 5). Initial pH in the salami in the drying room ranged from 5.28 (batch 1) to 5.58 (batch 5). During ripening, the pH decreased up to 5.3 in all batches except batch 5. However, after 10 weeks of ripening, the pH increased in all batches, reaching values ranging from 5.87 (batch 4) to 6.25 (batch 3) in the final product (Figure 5). 

Regarding water activity, a constant decrease was observed from values ranging from 0.953 (batch 6) to 0.982 (batch 3) in the salami in the drying room to values ranging from 0.842 (batch 3) to 0.884 (batch 5) in salami after 28 weeks of ripening (Figure 5). 

### 3.3. Generalized Linear Mixed Models

Generalized linear mixed models were created to explore whether variations in the production stage and the environment could account for some of the differences observed between batches in TBC and *Enterobacteriaceae* counts throughout the salami production process. The outcomes of this investigation are presented in Table 1 and Table 2.

Results from GLMMs indicated that the TBC in the drying, stuffing, and ripening phases were significantly higher than the TBC found in the meat mixture (*p* ≤ 0.05). Specifically, the ripening step produced the largest increase (4.33 ± 0.27 log cfu/g), which can be partially attributed to the increase in LAB populations. During ripening, a positive effect was predicted for TBC counts in 18- and 28-week ripened salami (Table 1). Regarding *Enterobacteriaceae* counts, there was a significant negative effect after mixing, indicating the decrease in microbial counts during the drying, stuffing, and ripening phases. A significant decrease in *Enterobacteriaceae* populations was predicted during salami ripening (Table 2). Significant negative effects were found between most of the analyzed surfaces and TBC and *Enterobacteriaceae* counts in the salami samples in the drying room, thus indicating an increase in microbial populations in the finished salami samples. Nevertheless, TBC counts from the water drainages (ripening and drying rooms) were similar to the TBC levels in the salami samples in the drying room (*p* > 0.05, Table 1). Regarding the inter-batch variability, random effects indicate a higher inter-batch variability for TBC in comparison to *Enterobacteriaceae*. 

### 3.4. Occurrence of Bacterial Pathogens

Regarding bacterial pathogens, none of the 480 samples collected were positive for VTEC or *Salmonella* spp. Four isolates were positive for *L. monocytogenes* and six for *S. aureus*, eight for *S. warneri*, one for *S. capitis*, and one for *S. xilosus*. Regarding *Enterobacteriaceae*, the following species were identified: *Klebsiella oxytoca* and *K. pneumoniae* (33), *E. coli* (30), *Citrobacter freundii* (26), *Enterobacter cloacae* (16), and *Routella planticola* (1) (Table S1). 

Bacteria were collected from mixed meat and salami during and at the end of the ripening period, and environmental swabs were gathered during production. In particular, pathogens were mostly found in the meat mixture (22), the surface of the table in the stuffing room (15), and in the salami in the drying room (25) and after 3 weeks of ripening (19) (Table S2). Following ripening, the number of pathogens decreased from 25 in salami in the drying room to 5 and 12 in salami at 18 and 28 weeks of ripening, respectively (Table S2). One *Enterobacter cloacae* strain, six *E. coli*, two *Citrobacter freundii*, and two *K. pneumoniae* were found in the finished product, suggesting that although the ripening process was effective in reducing the risk associated with the presence of bacterial pathogens, it was not enough to guarantee the lack of bacterial pathogens in the final food product. Although the water activity in the final product was lower than 0.90, data on the increased pH suggest the need to better control this parameter, for example, by considering the possibility of shortening the ripening period from 28 to 18 weeks.

## 4. Discussion

In the present study, the effect of pH and water activity on the number of bacterial indicators of the process’s hygiene and the occurrence of foodborne pathogens was assessed in an artisanal Italian production of organic salami. 

pH is an essential parameter in fermented meat products. The acid hurdle is crucial for the control of the safety of the product [38,39]. pH values below 5.3 are essential to inhibit the growth of Gram-positive bacteria such as *S. aureus*. The pH of the majority of Mediterranean-style fermented sausages is approximately 4.5/5.4, which has several beneficial effects on both the shelf life and the manufacturing process [39,40]. However, the final pH can rise up to 6.0/6.7 in some low-acid fermented sausages (e.g., Soudjouk, Fuet).

In the present study, the pH decreased below 5.3 after 10 weeks of ripening, after which an increase to values higher than 6 in the final products was achieved. These results might be associated with the lack of standardized starter cultures and the different growth rates of autochthonous LAB groups in each batch. In salami prepared with starter cultures of dairy origin, pH 5.3 was reached after 3 weeks of ripening [41]. Neutral values of pH in the final product might reduce the acid hurdles and thus enhance the growth of bacteria. In the present study, although the number of *Enterobacteriaceae* was lower than the detection limit in most final products, bacterial pathogens were detected. 

Water activity values lower than 0.90 and 0.86 are essential to control the growth of Gram-positive and Gram-negative bacterial pathogens, respectively [5,42]. In the final product of the present study, all batches showed a water activity value lower than 0.90, but only one (batch 3) fell under the 0.86 limit (batch 3, 0.842), suggesting a potential hurdle for *S. aureus* control. Although not found in the final product, *S. aureus* was found in raw materials, salami in the drying room, and salami after 3 weeks of ripening. This finding suggests the relevance of a high microbiological quality of raw materials, especially when pH and water activity hurdles cannot be fully maintained. 

Besides bacterial pathogens being found in the final product, the detection of pathogens in the processing environment is of concern since potential events of cross-contamination might occur involving both workers and the food product before commercialization. 

The ability of *L. monocytogenes* to survive on several environmental stress conditions boost the likelihood of detecting this foodborne pathogen in contaminated ready-to-eat products of meat, fish, and dairy-origin products [43,44]. Recently, several *L. monocytogenes* outbreaks were associated with ready-to-eat meat product contamination from Italy and worldwide [45,46,47], among which a recent US outbreak involved Italian-type salami, mortadella, and prosciutto [48]. Despite *L. monocytogenes* not being detected in the final products, it was found in the processing environment of batch 2 (manhole of the drying room). Therefore, a more intensive control of the contamination routes in the salami processing plant might be necessary. Likewise, *S. aureus* is able to tolerate a wide range of environmental conditions, including pH ranges from 4.5 to 9.0 and NaCl concentrations up to 9%. Many strains have been recently recovered from dry-cured meat processing facilities and related products [49,50]. The finding of six *S. aureus* strains from all batches but the first one, as well as from different sources (including meat mixture, processing surfaces, and in salami of the drying and ripening rooms), suggests that different strains have been introduced in the food processing chain through raw materials, hygiene failures, or food handlers. 

*Enterobacteriaceae* represented the predominant family recovered across the salami facility, resulting in 6 species and 106 strains confirmed overall (84% prevalence). Among *Enterobacteriaceae*, *E. coli* corresponded to the dominant species, exhibiting a remarkable predominance in the meat mixture and associated environment up to the drying and ripening of the product. Moreover, it represented the bacterial species isolated in the highest proportion from the final product (*n* = 6 strains). Additionally, *K. oxytoca* and *K. pneumoniae* as well as *C. freundii* and *E. cloacae* were recovered from five to six batches; these were associated with the environment and food matrices of the meat mixture and the drying room, then persisting in the salami throughout the ripening up to the final products, where few strains were detected. Though strains belonging to enterohaemorrhagic *E. coli* (EHEC) have been attributed to foodborne outbreaks associated with fermented sausage consumption [51], the persistence of commensal *Escherichia* spp. strains has been already pointed out in spontaneously fermented sausages of the Mediterranean area [52]. Correspondingly, *Klebsiella* spp. were detected from minced pork meat during fermentation and raw pork sausages before ripening in Belgium and Spain [53,54], as well as *E. cloacae* and *Citrobacter* spp. during the ripening of traditional fermented sausages [53,55]. Considering that these bacteria have already been involved in nosocomial infections [56,57,58], further investigations should be addressed to verify their potential role as contributors to food-related infections. 

Whether introduced from raw materials or environmental contamination due to poor hygienic conditions, enhanced hurdle technologies, disinfection measures, and training should be properly adopted to control the growth of potentially pathogenic or undesirable bacteria along the whole food chain. In the present study, the meat mixture and the surface of the table in the stuffing room were spotted as principal potential sources of contamination. The meat mixture might have been contaminated at the first steps of the production during the cutting and addition of spices, or before that, during the primary production. Unfortunately, no samples from pig carcasses were collected; therefore, no speculations can be formulated about carcass hygiene. However, since the same person owns both the primary production of the organic pig and the food production of organic salami, cross-contamination cannot be ruled out. 

The adoption of tailor-made biosecurity plans including the hygiene of farms and workers has been described as an effective measure to reduce the risk of occurrence of foodborne pathogens in pig farms and the dissemination of those bacteria to humans through direct contact [59]. In addition, special attention should be paid to hygiene procedures for surfaces in direct contact with food [60]. The sampling of surfaces in direct contact with food should be prioritized, and results obtained from different batches should be compared in order to identify deviations and take corrective actions [60]. Besides the implementation of hygiene procedures, food safety training has been described as particularly effective in small-scale facilities. In particular, food safety training programs, which incorporate both knowledge and behavior-based training, were described as the most effective in commercial food services [61].

## 5. Conclusions

High inter-batch variability was detected in the physicochemical and microbiological parameters of organic salami produced in an artisanal factory, confirming process standardization to be a challenge in small-scale, not fully automized, production facilities. Higher TBC in the meat mixture and on the surface of the table in the stuffing room were associated with a higher occurrence of bacterial pathogens, suggesting TBC to be a good predictor of the microbial quality of the final product. This predictor might be of particular relevance, especially when the protocol of production cannot guarantee the acid and desiccation hurdles essential for biohazard control. In these conditions, enhanced hygiene measures and training could be effective control measures against the growth of potentially pathogenic or undesirable bacteria along the whole food chain.

## Figures and Tables

**Table 1:** Estimations of model parameters in linear mixed models with random effects, investigating the influence of “Stage” and “Sample” variables on total bacteria counts (TBC) in Italian salami, alongside assessments of between-batch variability.

Model	Parameters	Estimate (SE)	t-Value	Pr > |t|
**Main effects: Stage ^1^**	**Random effects (σ)**			
	Batch in factory	0.504	-	-
	Residual	1.827	-	-
	**Fixed effects**			
	Intercept	3.153 (0.313)	10.085	<0.001
	Drying	2.941 (0.304)	9.674	<0.001
	Stuffing	0.845 (0.304)	2.780	0.005
	Ripening	4.327 (0.272)	15.916	<0.001
**Main effects: Sample ^2^**	**Random effects (σ)**			
	Batch in factory	0.503	-	-
	Residual	1.390	-	-
	**Fixed effects**			
	Intercept	7.256 (0.335)	21.615	<0.001
	Sample: MB	−3.783 (0.367)	−10.318	<0.001
	Sample: SBR_28	1.148 (0.367)	3.130	0.002
	Sample: SBR_18	1.117 (0.367)	3.047	0.002
	Sample: SBR_10	0.092 (0.367)	0.251	0.801
	Sample: SBR_3	0.992 (0.367)	2.705	0.007
	Sample: SEM	−4.421 (0.367)	−12.058	<0.001
	Sample: SED	−3.311 (0.367)	−9.031	<0.001
	Sample: SER	−1.796 (0.367)	−4.899	<0.001
	Sample: SM	−4.574 (0.367)	−12.473	<0.001
	Sample: STM	−3.681 (0.367)	−10.039	<0.001
	Sample: SWD	−0.173 (0.367)	−0.472	0.637
	Sample: SWM	−1.517 (0.367)	−4.136	<0.001
	Sample: SWR	−0.202 (0.367)	−0.550	0.583

^1^ TBC in mixing stage was set as reference category. ^2^ TBC in salami samples of drying room was set as reference category. MB: meat mixture; SEM: wall swab (stuffing room); SED: wall swab (drying room); SER: wall swab (ripening room); SM: minced-meat machine swab (stuffing room); STM: surface swab (stuffing room); SWD: water drainage swab (drying room); SWM: water drainage swab (stuffing room); SWR: water drainage swab (ripening room); SBR: ripened salami (3, 10, 18, and 28 weeks).

**Table 2:** Estimations of model parameters in linear mixed models with random effects, evaluating the impact of “Stage” and “Sample” variables on *Enterobacteriaceae* in Italian salami, and including assessments of between-batch variability.

Model	Parameters	Estimate (SE)	t-Value	Pr > |t|
**Main effects: Stage ^1^**	**Random effects (σ)**			
	Batch in factory	0.193	-	-
	Residual	1.654	-	-
	**Fixed effects**			
	Intercept	1.952 (0.228)	8.576	<0.001
	Drying	−0.588 (0.275)	−2.132	0.034
	Stuffing	−1.337 (0.275)	−4.849	<0.001
	Ripening	−0.658 (0.246)	−2.670	0.008
**Main effects: Sample ^2^**	**Random effects (σ)**			
	Batch in factory	0.503	-	-
	Residual	1.390	-	-
	**Fixed effects**			
	Intercept	4.095 (0.171)	24.010	<0.001
	Sample: MB	−0.190 (0.188)	−1.011	0.313
	Sample: SBR_28	−3.096 (0.188)	−16.506	<0.001
	Sample: SBR_18	−3.045 (0.188)	−16.233	<0.001
	Sample: SBR_10	−1.926 (0.188)	−10.267	<0.001
	Sample: SBR_3	−0.547 (0.188)	−2.909	0.004
	Sample: SEM	−4.095 (0.188)	−21.830	<0.001
	Sample: SED	−4.095 (0.188)	−21.830	<0.001
	Sample: SER	−4.095 (0.188)	−21.830	<0.001
	Sample: SM	−4.095 (0.188)	−21.830	<0.001
	Sample: STM	−2.247 (0.188)	−11.978	<0.001
	Sample: SWD	−4.095 (0.188)	−21.830	<0.001
	Sample: SWM	−4.095 (0.188)	−21.830	<0.001
	Sample: SWR	−4.095 (0.188)	−21.830	<0.001

^1^ *Enterobacteriaceae* in mixing stage was set as reference category. ^2^ *Enterobacteriaceae* in salami samples in the drying room was set as reference category. MB: meat mixture; SEM: wall swab (stuffing room); SED: wall swab (drying room); SER: wall swab (ripening room); SM: minced-meat machine swab (stuffing room); STM: surface swab (stuffing room); SWD: water drainage swab (drying room); SWM: water drainage swab (stuffing room); SWR: water drainage swab (ripening room); SBR: ripened salami (3, 10, 18, and 28 weeks).

## Data Availability

The original contributions presented in the study are included in the article. Further inquiries can be directed to the corresponding authors.

## Supplementary Materials

The following supporting information can be downloaded at: https://www.mdpi.com/article/10.3390/foods12224086/s1, Table S1: Results of the species identification of bacterial food-borne pathogens and pathogens of clinical importance from the 126 isolates in the six tested batches (B1–B6); Table S2: Results of the species identification of bacterial food-borne pathogens and pathogens of clinical importance from the 126 isolates in relation to the type of sample.
