# Supplementary material for: Variability in Physicochemical Parameters and Its Impact on Microbiological Quality and Occurrence of Foodborne Pathogens in Artisanal Italian Organic Salami
Source: Foods. 2023 Nov 10;12(22):4086. doi: 10.3390/foods12224086 (PMC10670534; doi:10.3390/foods12224086)
Supplement: Supplementary file 1 [file foods-12-04086-s001.zip › foods-2675243-supplementary.pdf]

Table S1 – Results of the species identification of bacterial food-borne pathogens and pathogens of clinical importance from the 126 isolates in the six tested batches (B1-B6).

Species	B1	B2	B3	B4	B5	B6	Total
Listeria monocytogenes	0	4	0	0	0	0	4
Staphylococcus aureus	0	2	1	1	1	1	6
Escherichia coli	4	6	6	5	5	4	30
Klebsiella pneumoniae	5	2	2	0	8	3	20
Klebsiella oxytoca	1	3	4	1	1	3	13
Raoultella planticola	0	0	0	0	0	1	1
Citrobacter freundii	4	4	5	2	7	4	26
Enterobacter cloacae	2	0	5	3	2	4	16
Staphylococcus warneri	0	4	0	1	1	2	8
Staphylococcus capitis	0	0	0	0	0	1	1
Staphylococcus xilosus	1	0	0	0	0	0	1
Total	17	25	23	13	25	23	126

Table S2 – Results of the species identification of bacterial food-borne pathogens and pathogens of clinical importance from the 126 isolates in relation to the type of sample.

Bacterial Pathogen	MB	SEM	SWM	STM	SM	SBD	SED	SWD	SBR	SER	SWR	SBR10	SBR18	SBR28	Total
L. monocytogenes								4							4
S. aureus	2			1		2			1						6
E. coli	5		1	3	2	4			4			2	3	6	30
K. pneumoniae	9		2		1	3		1	1			1		2	20
K. oxytoca	1		1	2		5			3			1			13
Routella planticola						1									1
C. freundii	1		1	3		5		3	6			2	2	3	26
E. cloacae	1		2	3	1	2			3			3		1	16
S.warneri	3			2		3									8
S.capitis				1											1
S.xilosus									1						1
Total	22	0	7	15	4	25	0	8	19	0	0	9	5	12	126

MB: meat mixture; SEM: wall swab - stuffing room, SWM: manhole swab - stuffing room; STM: surface swab - stuffing room; SM: minced meat machine swab -stuffing room; SBD: salami in drying room, SED: wall swab - drying room; SWD: manhole swab - drying room; SBR: salami - 3 weeks of ripening; SER: wall swab - ripening room; SWR: manhole swab - ripening room; SBR10: salami - 10 weeks of ripening, SBR18: salami - 18 weeks of ripening; SBR28: salami - 28 weeks of ripening.
